# Left ventricular endocardial pacing is less arrhythmogenic than conventional epicardial pacing when pacing in proximity to scar

**DOI:** 10.1016/j.hrthm.2020.03.021

**Published:** 2020-08

**Authors:** Caroline Mendonca Costa, Aurel Neic, Karli Gillette, Bradley Porter, Justin Gould, Baldeep Sidhu, Zhong Chen, Mark Elliott, Vishal Mehta, Gernot Plank, C.A. Rinaldi, Martin J. Bishop, Steven A. Niederer

**Affiliations:** ∗ King’s College London, London, United Kingdom; † Medical University of Graz, Graz, Austria; ‡ Guy’s and St. Thomas’ Hospital, London, United Kingdom

**Keywords:** Cardiac resynchronization therapy, Dispersion of repolarization, Infarct scar, Patient-specific modeling, Ventricular tachycardia

## Abstract

**Background:**

Epicardial pacing increases risk of ventricular tachycardia (VT) in patients with ischemic cardiomyopathy (ICM) when pacing in proximity to scar. Endocardial pacing may be less arrhythmogenic as it preserves the physiological sequences of activation and repolarization.

**Objective:**

The purpose of this study was to determine the relative arrhythmogenic risk of endocardial compared to epicardial pacing, and the role of the transmural gradient of action potential duration (APD) and pacing location relative to scar on arrhythmogenic risk during endocardial pacing.

**Methods:**

Computational models of ICM patients (n = 24) were used to simulate left ventricular (LV) epicardial and endocardial pacing 0.2–3.5 cm from a scar. Mechanisms were investigated in idealized models of the ventricular wall and scar. Simulations were run with/without a 20-ms transmural APD gradient in the physiological direction and with the gradient inverted. Dispersion of repolarization was computed as a surrogate of VT risk.

**Results:**

Patient-specific models with a physiological APD gradient predict that endocardial pacing decreases VT risk (34%; *P* <.05) compared to epicardial pacing when pacing in proximity to scar (0.2 cm). Endocardial pacing location does not significantly affect VT risk, but epicardial pacing at 0.2 cm compared to 3.5 cm from scar increases it (*P* <.05). Inverting the transmural APD gradient reverses this trend. Idealized models predict that propagation in the direction opposite to APD gradient decreases VT risk.

**Conclusion:**

Endocardial pacing is less arrhythmogenic than epicardial pacing when pacing proximal to scar and is less susceptible to pacing location relative to scar. The physiological repolarization sequence during endocardial pacing mechanistically explains reduced VT risk compared to epicardial pacing.

## Introduction

Endocardial pacing has been shown to improve response to cardiac resynchronization therapy (CRT) in comparison to conventional left ventricular (LV) epicardial pacing^1^^,^^2^ due to access to fast endocardial conduction (FEC).^3^ Epicardial pacing reverses the physiological sequence of activation and repolarization, which is known to increase dispersion of repolarization and facilitate arrhythmias.^4^ Endocardial pacing may be less arrhythmogenic than epicardial pacing, as it preserves the physiological sequences of activation and repolarization.

Our previous study predicted that conventional epicardial LV pacing in proximity to scar increases repolarization gradients, which in turn increases ventricular tachycardia (VT) risk by increasing the vulnerable window for unidirectional block.^5^ Endocardial pacing increases the area accessible for lead implantation because it is not constrained by coronary sinus anatomy, allowing operators to target lead position based on the individual’s anatomy and scar.^6^^,^^7^ This enables pacing at an optimal location to maximize response while avoiding increasing VT risk. However, susceptibility to arrhythmogenesis during endocardial pacing has not been systematically investigated, and the role of pacing location relative to scar during endocardial pacing currently is unknown.

The primary aim of this study was to investigate the relative arrhythmogenic risk of endocardial pacing compared with epicardial pacing. We also investigated the role of pacing location relative to scar during endocardial pacing, as done previously for epicardial pacing,^5^ and the role of the direction of transmural propagation during endocardial and epicardial pacing on VT risk. We used a virtual cohort of patient-specific computational models of LV anatomy, scar, and border zone (BZ) to run electrophysiological (EP) simulations and compute dispersion of repolarization as a surrogate for VT risk.^5^

## Methods

### Models of patient-specific anatomy

**Figure 1:**
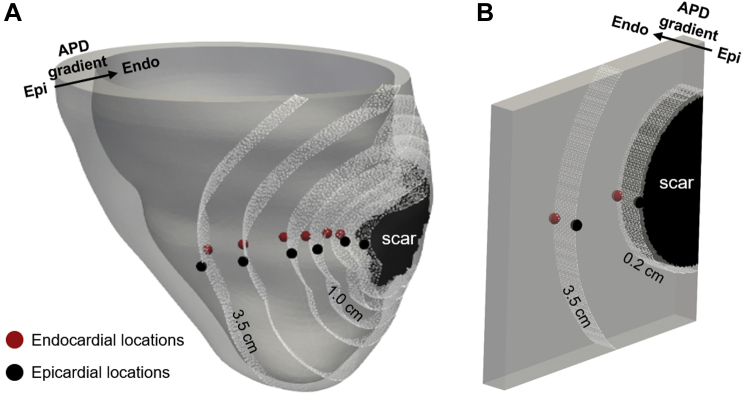
Pacing locations. Endocardial pacing locations are in *red* and epicardial pacing locations in *black.* Distances from the scar surface are indicated by the *white point cloud.* Epicardial (epi) and endocardial (endo) surfaces and the transmural action potential duration (APD) gradient are indicated by the *black arrow.***A:** Patient-specific model. Locations were chosen at (from *right* to *left*) 0.2, 0.5, 1.5, 2.5, and 3.5 cm from the scar surface. **B:** Idealized model. Locations were chosen at 0.2 and 3.5 cm from the scar surface.

We used 24 image-based patient-specific models of LV anatomy and scar morphology, as described previously.^5^ In brief, LV endocardium and epicardium contours were manually drawn in each short-axis slice of late gadolinium enhanced magnetic resonance imaging. Scar and BZ were segmented and reconstructed in 3 dimensions (3D). A finite element tetrahedral mesh (mean edge length 0.8 mm) was generated, and 3D-reconstructed scar and BZ segmentations^8^ were mapped onto it. Rule-based fibers were assigned to the models.^9^ An example is shown in Figure 1A. (These models are available online at http://doi.org/10.18742/RDM01-570.)

### Idealized models

We created idealized models of a ventricular wall wedge to investigate the role of transmural action potential duration (APD) gradient direction relative to pacing location (endocardium or epicardium) independent of the effects of ventricle anatomy. A 10- × 10- × 1-cm^3^ mesh of tetrahedral elements was created (mean edge length 800 μm). LV fiber orientations were assigned using a rule-based method.^9^ A circumferential and transmural scar with radius of 1.5 cm and a 0.2-cm-thick BZ were included on the left side of the mesh (Figure 1B).

### Selecting pacing locations

Pacing locations were selected on the endocardial and epicardial LV surfaces transmurally opposite to each other. For the patient-specific models, pacing locations were selected at 0.2, 0.5, 1.5, 2.5, and 3.5 cm from scar (Figure 1A), and at 0.2 and 3.5 cm (Figure 1B) for the idealized model. Distances from scar were computed using eikonal simulations.^5^

### FEC layer

**Figure 2:**
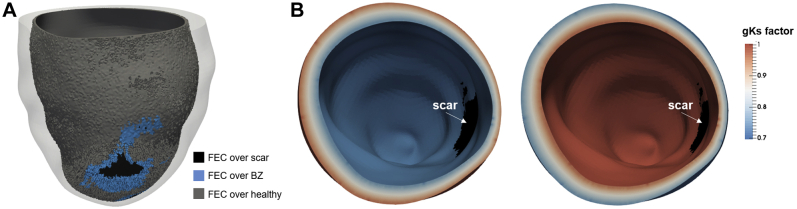
**A:** Example of a 1-mm-thick layer of fast endocardial conduction (FEC) over scar *(black),* border zone (BZ) *(blue),* and healthy tissue *(gray).***B:** Multiplying factor of the slow rectifying potassium current conductance (gKs) across the ventricular wall. Shown are examples of the physiological *(right)* and inverted *(left)* transmural gradient.

The presence of FEC is thought to improve response to endocardial CRT.^3^ Using the transmural coordinate of the universal ventricular coordinates system,^10^ we selected a 1-mm-thick FEC layer^3^ in each anatomic model. This layer was selected within the entire endocardial surface, including healthy, BZ, and scar tissue (Figure 2A).

### EP models and parameters

Activation and repolarization sequences were simulated, as in our previous study.^5^ In brief, the reaction-eikonal model^11^ coupled to the ten Tusscher model^12^ of human ventricular action potential were used, and activation was initiated at each pacing location. Transversely isotropic conduction velocities (CVs) of 0.67 and 0.3 m/s^13^ were prescribed to healthy tissue in the longitudinal and transverse directions, respectively. An isotropic CV of 0.15 m/s was prescribed to the BZ,^14^ and the scar core was modeled as nonconducting.

To the best of our knowledge, no CV measurements within an FEC layer in the presence of an infarct scar are currently available in the literature. Based on CV measurements within an FEC layer^15^ and BZ,^14^ we created 6 different FEC setups, in which a 2× faster CV was prescribed to the FEC layer over healthy tissue along the fiber direction,^15^ and an isotropic CV 2× faster than either healthy or BZ tissue was prescribed to the FEC layer over BZ/scar. The individual setups are detailed in Supplemental Table S1. Unless otherwise stated, results are shown with an FEC layer over healthy and BZ tissue, with a 2× faster CV over healthy tissue along the fiber direction, and an isotropic CV 2× faster than BZ tissue (setup 4 in Supplemental Table S1).

To investigate the role of transmural APD heterogeneity on arrhythmogenesis, a linear change in transmural APD of 20 ms was implemented across the ventricular wall in line with previous measurements of transmural APD heterogeneity in heart failure (HF).^16^ This was achieved by multiplying the conductance of the slow rectifying potassium current gK_s_ by a factor of 0.7 to 1 giving an APD of 280 to 260 ms, respectively, and reflecting a 20-ms APD gradient.^16^ An inverted gradient was also implemented. An example of the transmural APD gradients is shown in Figure 2B.

Results are presented with the patient-specific models for a control model (20-ms APD gradient in the physiological direction), a model with no APD gradient, and a model with an inverted APD gradient (opposite to physiological direction). All model results presented include an FEC layer as in setup 4 of Supplemental Table S1. Results with different FEC setups are shown in the Supplemental Figure S1.

### Computing dispersion of repolarization

We used the volume of high repolarization gradients (HRGs) within 1 cm around the scar as a metric of local dispersion of repolarization and a surrogate for arrhythmogenic risk, as done previously.^5^ In brief, repolarization times, local repolarization gradients, and the volume of tissue with repolarization gradients above a threshold of 3 ms/mm^17^ were computed.

### Statistical analysis

Balanced 1-way analysis of variance with Tukey-Kramer *post hoc* tests were used to compare the HRG volume between the patient-specific pacing locations. Paired Student *t* tests were used to compare HRG volume between endocardial and epicardial pacing at each pacing location. Quantitative results are shown as standard bar plots including error bars, which describe the standard variation of values within the 24 patient models. *P* <.05 was considered significant.

## Results

### Pacing location and modality

**Figure 3:**
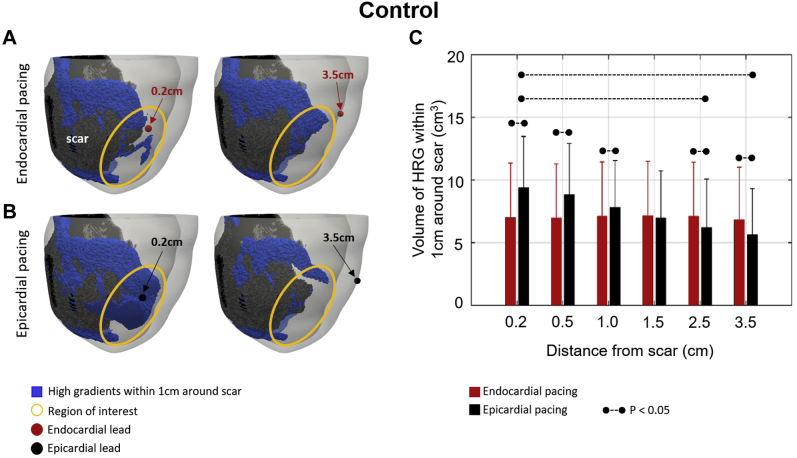
Control case with fast endocardial conduction and physiological transmural action potential duration gradient. **A, B:** High repolarization gradients (HRGs) within 1 cm around the scar *(blue)* for endocardial **(A)** and epicardial **(B)** pacing. Endocardial lead locations are shown by *red circles* and epicardial lead locations by *black circles.* Regions of interest are highlighted by *yellow circles.***C:** HRG volume for endocardial (*red*) and epicardial (*black*) pacing at 0.2–3.5 cm from a scar. *Dashed lines* indicate a significant difference (*P* <.05).

We computed repolarization gradients and HRG volume using our control model. Figure 3C shows a significant reduction in HRG volume when pacing away from the scar for epicardial (black) but not for endocardial (red) pacing. Specifically, HRG volume is significantly smaller when pacing at 3.5 cm than 0.2 cm from the scar during epicardial pacing. HRG volume at 0.2–1 cm is significantly smaller (*P* <.05) during endocardial compared to epicardial pacing, significantly larger (*P* <.05) at 2.5–3.5 cm during endocardial compared to epicardial pacing, and similar at 1.5 cm. This is illustrated in Figures 3A and 3C, which show an example of the spatial distribution of HRG (blue) during endocardial (Figure 3A) and epicardial (Figure 3B) pacing. The difference in HRG volume between endocardial and epicardial pacing is particularly evident when focusing on the highlighted regions within the yellow circles, with a visibly larger reduction in the blue HRG volumes when pacing 3.5 cm compared to 0.2 cm from the scar for epicardial than for endocardial pacing.

### Transmural APD gradients

**Figure 4:**
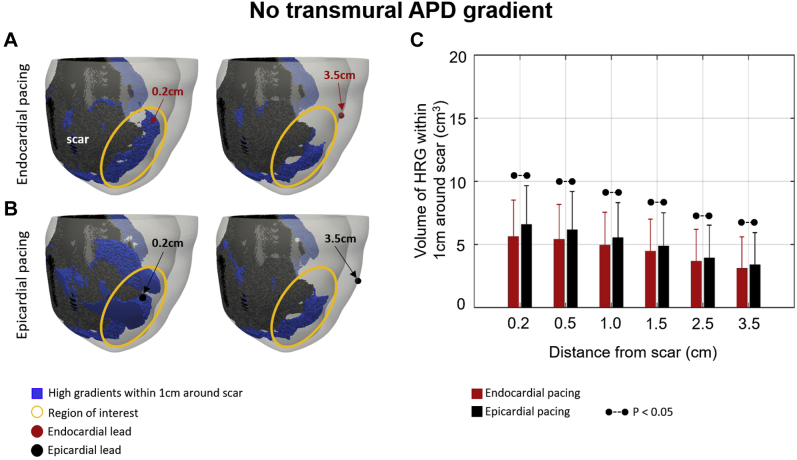
No transmural action potential duration (APD)gradient case. **A, B:** High repolarization gradients (HRGs) within 1 cm around the scar *(blue)* for endocardial **(A)** and epicardial **(B)** pacing. Endocardial lead locations are shown by *red circles* and epicardial lead locations by *black circles.* Regions of interest are highlighted by *yellow circles.***C:** HRG volume for endocardial *(red)* and epicardial *(black)* pacing at 0.2–3.5 cm from a scar. *Dashed lines* indicate a significant difference (*P*<.05).

**Figure 5:**
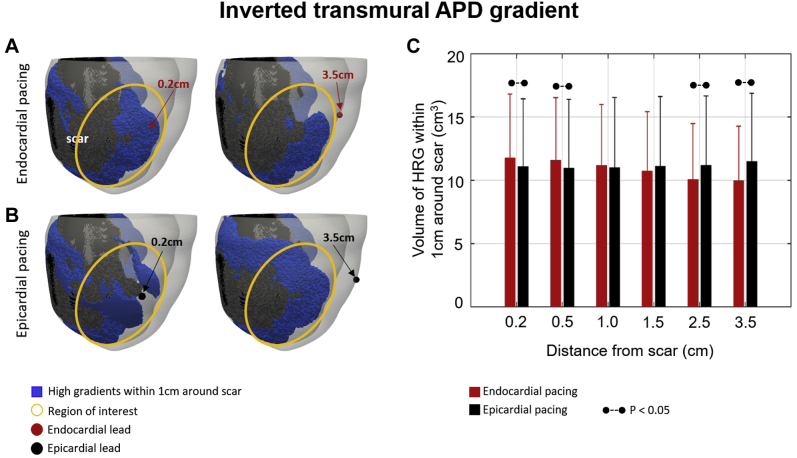
Inverted transmural action potential duration (APD) gradient case. **A, B:** High repolarization gradients (HRGs) within 1 cm around the scar *(blue)* for endocardial **(A)** and epicardial **(B)** pacing. Endocardial lead locations are shown by *red circles* and epicardial lead locations by *black circles.* Regions of interest are highlighted by *yellow circles.***C:** HRG volume for endocardial *(red)* and epicardial *(black)* pacing at 0.2–3.5 cm from a scar. *Dashed lines* indicate a significant difference (*P* <.05).

Using the patient models, we found that in simulations without a transmural APD gradient (Figure 4), endocardial (Figure 4A) and epicardial (Figure 4B) pacing show similar results with a trend toward reduced HRG volume when pacing away from a scar (Figure 4C), although not significant. Inverting the direction of the APD gradient creates a smaller HRG volume when pacing at the endocardial surface (Figures 5A and 5C) at 3.5 cm than at 0.2 cm from a scar. This is similar to what is observed for epicardial pacing in the control case (Figures 3B and 3C), although the difference is not significant (Figure 5C). The HRG volume is significantly larger at 0.2 cm during endocardial compared to epicardial pacing and significantly smaller at 3.5 cm. Focusing on the highlighted regions indicated by the yellow circles (Figures 5A and 5B), a slightly smaller HRG volume is observed when pacing 3.5 cm compared to 0.2 cm from the scar during endocardial (Figure 5A) pacing, whereas the opposite is observed during epicardial (Figure 5B) pacing.

### FEC

To investigate the impact of the morphological and functional properties of the FEC layer over scar and BZ, we created 6 different setups, including no FEC layer, FEC over healthy and BZ only, and FEC over scar with varying CVs (Supplemental Table S1). Epicardial pacing created smaller HRG volumes when pacing away from scar than in proximity to it, and endocardial pacing was not sensitive to pacing location relative to scar across all setups (Supplemental Figure S1). Overall, the presence of FEC (setups 2 to 6) reduced the mean HRG volume at a given pacing location compared to no FEC (setup 1). The level of statistical significance across different setups varied, with setup 6 (FEC over scar with CV 2× the CV of the BZ) showing no significant difference between HRG volume for epicardial pacing locations and larger variability across models, as evidenced by larger error bars compared to the other setups.

### Idealized models

**Figure 6:**
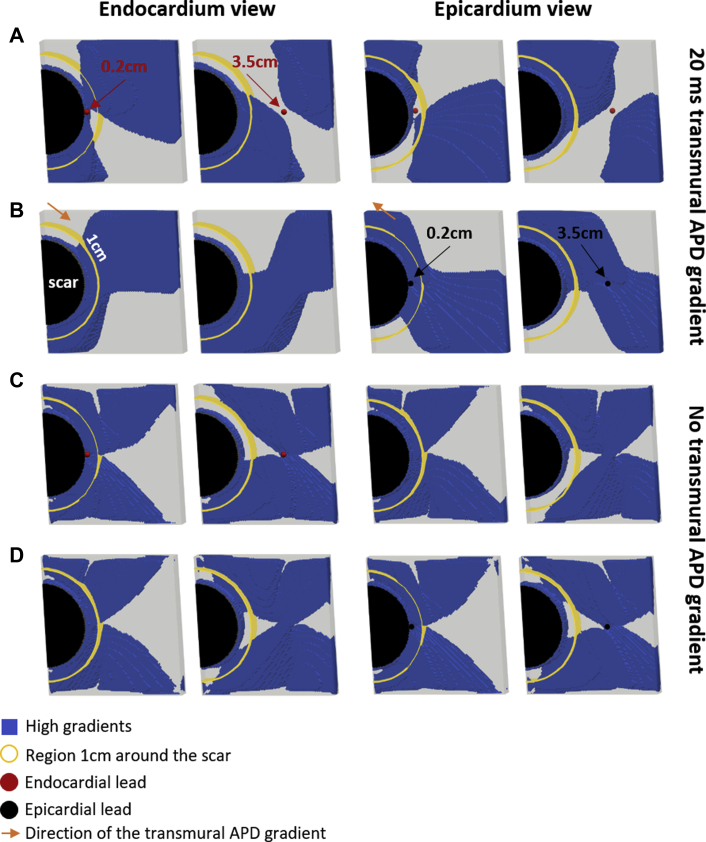
Idealized models. High repolarization gradients (blue) for endocardial **(A, C)** and epicardial **(B, D)** pacing. Shown are epicardial and endocardial views when pacing 0.2 and 3.5 cm from scar. Endocardial lead locations are shown by *red spheres* and epicardial lead locations by *black spheres*. Region 1 cm around the scar is highlighted by *yellow circles. Orange arrows* indicate the direction of the action potential duration (APD) gradient across the wall.

To demonstrate that these are general findings, independent of the patient-specific anatomies, we ran additional simulations using our idealized model. Consistent with the control models, we found that pacing 0.2 cm from the scar (Figure 6B) creates a 1.52× larger volume of HRG within 1 cm around the scar (yellow circle) during epicardial compared to endocardial pacing (Figure 6A). Conversely, pacing at 3.5 cm creates a 0.85× smaller HRG volume during epicardial compared to endocardial pacing. Pacing 3.5 cm instead of 0.2 cm from the scar creates a substantially smaller HRG volume during epicardial pacing (38% decrease). Conversely, pacing 3.5 cm compared to 0.2 cm from the scar creates a slightly larger (11%) HRG volume during endocardial pacing. These 2 findings are comparable with the patient models, in which a significant change in HRG volume is observed during epicardial pacing but not during endocardial pacing.

To further confirm our findings, we ran simulations using the idealized models without a transmural APD gradient and paced at the “endocardium” (Figure 6C) and “epicardium” (Figure 6D) surfaces 0.2 and 3.5 cm from the scar. HRG volume in this case is virtually identical for endocardial and epicardial pacing, and pacing 0.2 cm from the scar creates a larger HRG volume compared to pacing 3.5 cm during endocardial (52%) and epicardial (54%) pacing. This differs from the patient models without an APD gradient (Figure 4), in which HRG volume is significantly smaller during endocardial compared to epicardial pacing. This is illustrated in Supplemental Figure S2 and is consistent with a larger volume of viable tissue at the epicardium in the patient models (Supplemental Figure S3), which leads to a larger HRG volume (Supplemental Figure S4) at the epicardium than at the endocardium.

**Figure 7:**
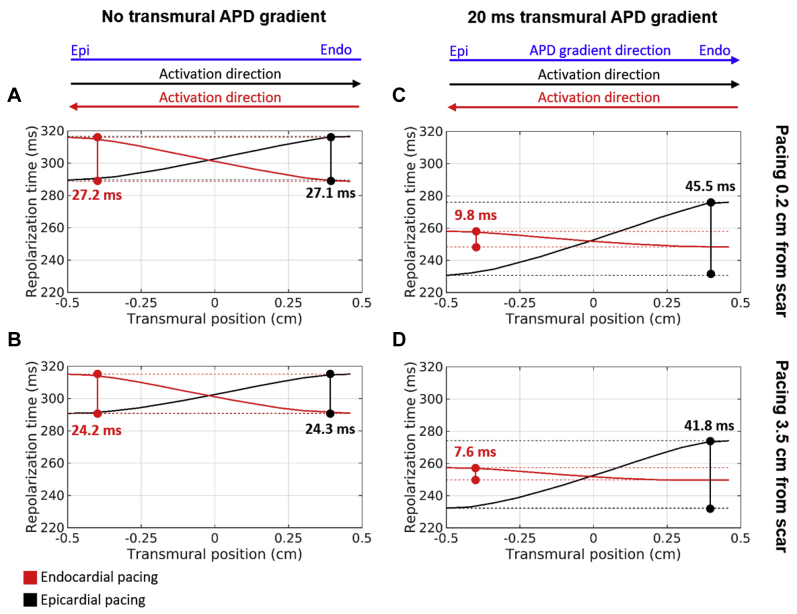
Repolarization times in the transmural direction across the ventricular wall of the idealized models when pacing at endocardial *(red)* and epicardial *(black)* surfaces. Shown are results with **(C, D)** and without **(A, B)** a transmural action potential duration (APD) gradient when pacing 0.2 cm **(A, C)** and 3.5 cm **(B, D)** from the scar. *Dashed lines* indicate the maximum and minimum repolarization times. The direction of activation during endocardial and epicardial pacing as well as the direction of the transmural APD gradient *(blue)* are indicated at the top.

We also investigated the change in repolarization times within the wall in the transmural direction in the absence (Figures 7A and 7B) and presence (Figures 7C and 7D) of a transmural APD gradient. In the absence of a transmural APD gradient (Figures 7A and 7B), transmural repolarization times increase in the direction of activation for both endocardial and epicardial pacing and with a similar transmural dispersion of repolarization (24.2–27.2 ms) for both pacing modalities and locations (0.2 and 3.5 cm). In the presence of a transmural APD gradient (Figures 7C and 7D), transmural repolarization times also increase in the direction of activation; however, they increase 4.6–5.5× more during epicardial compared to endocardial pacing. Compared to no gradient, repolarization times increase by ∼40% during epicardial pacing and decrease by ∼35% during endocardial pacing. Transmural dispersion of repolarization decreases when pacing at 0.2 compared to 3.5 cm in all cases, but the difference is small (2.2–3.8 ms).

## Discussion

Our main finding was that endocardial pacing is less arrhythmogenic than epicardial pacing when pacing in proximity to scar. Pacing at the endocardial surface, where APD is longest, provides a mechanistic explanation for this decreased risk during endocardial pacing. The presence and morphological properties of an FEC layer did not substantially affect our findings.

### Mechanisms of decreased VT risk during endocardial pacing

Under physiological conditions, endocardial cells have a longer APD than epicardial cells. This characteristic is responsible for synchronizing repolarization and creating a positive T wave on electrocardiogram.^18^ In HF, this transmural APD gradient is reduced compared to healthy conditions,^16^ but a substantial (∼20 ms) APD gradient across the wall persists. During epicardial pacing, the physiological direction of repolarization (from epicardium to endocardium) is reversed, increasing transmural dispersion of repolarization and arrhythmia risk.^4^ Conversely, the physiological direction of repolarization is preserved during endocardial pacing, suggesting it may be less arrhythmogenic than epicardial pacing.

We investigated the role of the presence and direction of the transmural APD gradient relative to the direction of activation on HRG volume. Our simulations using idealized computational models predict that propagation from the surface with longest APD to shortest APD, as is the case during endocardial pacing, attenuates the repolarization gradients due to pacing (Figure 6A). This phenomena can be explained by decreased electrotonic load of repolarization during endocardial pacing, as epicardial cells repolarize faster than endocardial cells, thus decreasing (∼35%) the total transmural repolarization time (Figure 7B) in comparison with the case without an APD gradient (Figure 7A). Conversely, propagation in the same direction of the APD gradient, as is the case during epicardial pacing, increases the electrotonic load for repolarization and total transmural repolarization time (∼40%), thus exacerbating the repolarization gradients created due to pacing and creating a larger HRG volume in the vicinity of the scar (Figure 6B).

As the effect of pacing on HRG is attenuated during endocardial pacing due to pacing at the surface with the longest APD, the impact of pacing location relative to scar is decreased, and no substantial change in the HRG volume when pacing in proximity and away from scar is observed in the simulations with both idealized (Figure 6A) and patient-specific (Figure 3) models. Conversely, pacing in proximity to, instead of away from, scar creates larger HRG volumes during epicardial pacing (Figure 3), in agreement with our previous study.^5^

The trend toward decreased HRG volume when pacing away from a scar in the absence of a transmural APD gradient is similar during endocardial and epicardial pacing in both idealized (Figures 6C and 6D) and patient-specific (Figure 4C) models, although HRG volumes during endocardial and epicardial pacing differ significantly in the patient models. This is explained by the presence of a larger HRG volume close to the pacing surface than at the opposite surface and a larger volume of viable tissue at the epicardium due to a larger surface area and less scar. This allows the HRG created by pacing to expand into more tissue during epicardial pacing than during endocardial pacing (for details, see Section 2 in the Supplemental Material.) Moreover, the fact that the trend in HRG volume for epicardial and endocardial pacing is reversed when the transmural APD gradient is inverted in the patient-specific models (Figure 5) further demonstrates that it is the presence and direction of the transmural APD gradient relative to the direction of propagation that drives the HRG volumes in the vicinity of the scar created during endocardial and epicardial pacing.

### FEC

Conduction is ∼2 times faster at the endocardium than in the remaining myocardium,^15^ and access to FEC is associated with better resynchronization during endocardial pacing compared to epicardial pacing.^3^ A thin layer of tissue is known to survive at the subendocardium after infarction.^19^ However, to the best of our knowledge, whether FEC is preserved within this thin layer of tissue is currently not known. This surviving subendocardium layer is thin (<800 μm^19^), discontinuous, and with fibrosis^20^ and fiber disarray.^20^^,^^21^ Thus, it is unlikely to play a major role in activation and repolarization during endocardial pacing.^14^

We investigated the impact of the morphological and functional properties of the FEC layer over scar and BZ. Our simulations predict that the presence of FEC reduces the mean HRG volume compared to no FEC (Supplemental Figure S1). This finding was consistent across all setups, although the level of statistical significance varied. Of note, our sample size is relatively small, and the introduction of additional EP heterogeneity may increase variability between models and affect statistical significance.

### Comparison with other studies

Our finding that pacing in opposition to the physiological direction of propagation during epicardial pacing increases transmural dispersion of repolarization (Figure 7) is in agreement with a previous clinical study showing increased transmural dispersion of repolarization,^4^ prolonged QT interval, and increased arrhythmia risk during epicardial pacing. However, transmural dispersion of repolarization has been shown to decrease over time in patients who respond to CRT due to reverse remodeling.^22^ This is likely to reduce HRG volume and arrhythmogenic risk due to pacing in responders.

Although access to FEC has been shown to improve synchronization during endocardial pacing,^1^^,^^3^ it has not been associated with decreased arrhythmogenic risk. Our results show that the presence of FEC leads to faster activation/repolarization, which in turn decreases HRG volume and relative arrhythmogenic risk (Supplemental Figure S1). Our simulation results show that specific morphological and functional properties of the FEC layer may influence the final HRG volume created during both endocardial and epicardial pacing to a limited extent; however, experimental or clinical evidence is currently lacking.

### Clinical implications and limitations

The clinical use of endocardial pacing instead of epicardial pacing has increased in the past decades and has shown promising results.^2^^,^^23^ However, LV endocardial pacing is not without risks. When using a lead to deliver the endocardial stimulus there is increased thromboembolic risk^24^ and mitral valve impairment,^25^ whereas in a leadless system there is the risk of electrode embolization and the need to implant a separate ultrasound transmitter.^23^ In addition, all proposed endocardial pacing systems require retrograde arterial access^23^ or transseptal puncture,^24^ which can lead to complications. Moreover, the indications for endocardial pacing are still evolving. Currently, patients are often recruited if they cannot receive or do not respond to conventional CRT.^23^^,^^24^^,^^26^^,^^27^

Despite its limitations, endocardial pacing offers a feasible and attractive alternative to conventional epicardial pacing in patients with ischemic cardiomyopathy–HF, as it allows pacing at optimal locations for resynchronization^6^ while avoiding pacing in proximity to scar and increasing VT risk.^5^ Lead guidance^2^^,^^6^ indicating that the optimal lead location for epicardial pacing is in the vicinity of scar may also be an indication for an endocardial device, given reduced sensitivity to pacing location relative to scar on arrhythmogenic risk during endocardial pacing.

## Conclusion

Our study showed that endocardial pacing is less arrhythmogenic than epicardial pacing when pacing in proximity to scar in patients with ischemic cardiomyopathy–HF. This behavior is explained by the presence and direction of transmural APD gradients during HF. The beneficial effect of endocardial pacing on repolarization gradients is slightly enhanced by the presence of FEC.
