# Supplementary material for: Left ventricular endocardial pacing is less arrhythmogenic than conventional epicardial pacing when pacing in proximity to scar
Source: Heart Rhythm. 2020 Aug;17(8):1262–70. doi: 10.1016/j.hrthm.2020.03.021 (PMC7397521; doi:10.1016/j.hrthm.2020.03.021)
Supplement: Supplementary data [file mmc1.docx]

**Left ventricular endocardial pacing is less arrhythmogenic than conventional epicardial pacing when pacing in proximity to scar**

Caroline Mendonca Costa, Aurel Neic, Karli Gillette, Bradley Porter, Justin Gould, Baldeep Sidhu, Zhong Chen, Mark Elliott, Vishal Mehta, Gernot Plank, Christopher A. Rinaldi, Martin J. Bishop, and Steven A. Niederer

**Supplemental material**

1. **Fast endocardial conduction layer**

Conduction velocity within the fast endocardial conduction (FEC) layer is ~2 times faster along the fibre direction^1^. However, in the presence of an infarct scar, it is currently not known whether a FEC layer would overlap the scar and BZ or would be limited to the healthy portion of the heart. The functional properties of a FEC layer overlapping the scar/BZ are also not known. Thus, we modelled six different FEC setups, which are listed in Table S1. CV within the FEC layer was set as 2x the value of the CV within healthy or BZ tissue, as described in Table S1.

| **Setup** | **FEC over healthy** | **CV FEC over healthy** | **FEC over BZ** | **CV FEC over BZ** | **FEC over scar** | **CV FEC over scar** |
| --- | --- | --- | --- | --- | --- | --- |
| 1 | no | -- | no | -- | no | -- |
| 2 | yes | 2 x healthy | no | -- | no | -- |
| 3 | yes | 2 x healthy | yes | 2 x healthy | no | -- |
| 4 | yes | 2 x healthy | yes | 2 x BZ | no | -- |
| 5 | yes | 2 x healthy | yes | 2 x healthy | yes | 2 x healthy |
| 6 | yes | 2 x healthy | yes | 2 x BZ | yes | 2 x BZ |

Table S1: Fast endocardial conduction (FEC) layer setups. Each pair of columns indicates the location of the FEC layer and its respective conduction velocity (CV).

We investigated the impact of different functional and morphological properties of a FEC layer on the volume of HRG during endocardial and epicardial pacing. Simulations were run for the FEC setups described in Table S1. A 20ms (HF) transmural APD gradient was used. Figure S1 shows a trend towards smaller volumes of HRG when pacing away from the scar for epicardial but not endocardial pacing in all FEC setups. The difference between the volume of HRG when pacing 0.2cm and 3.5cm from the scar is significant (P<0.05) for all setups except setup 6.


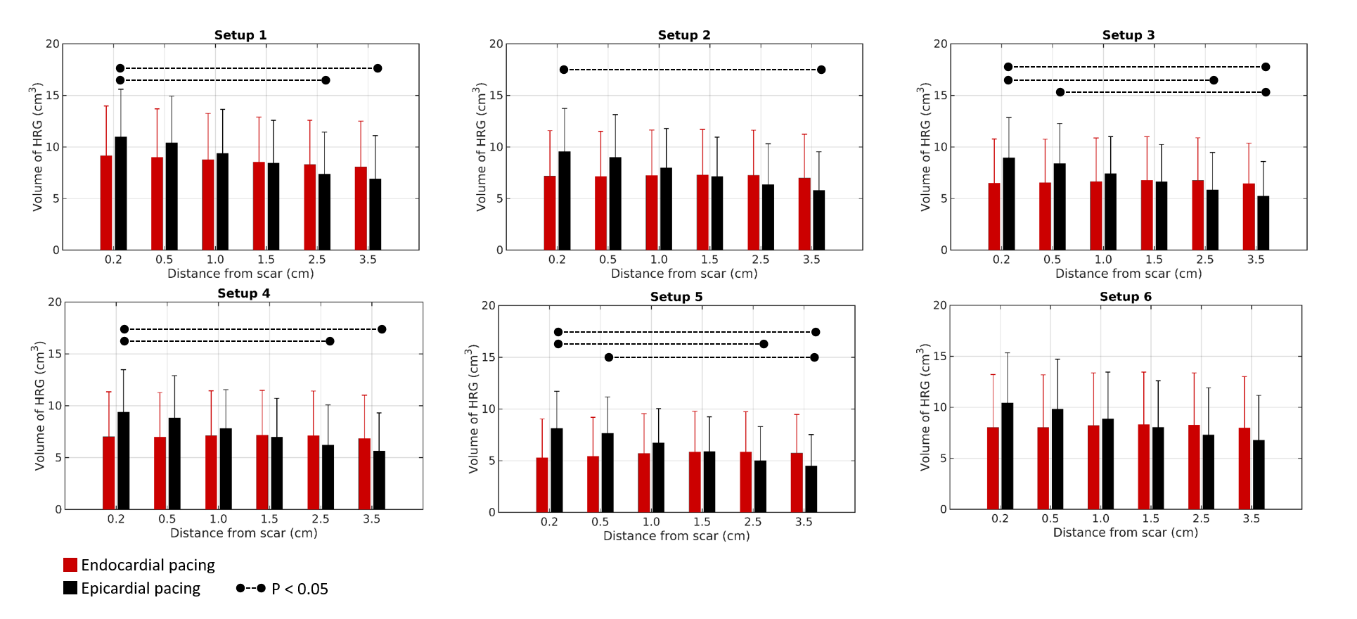


Figure S1: Role of fast endocardial conduction (FEC) on the volume of high repolarization gradients (HRG) during endocardial (red) and epicardial (black) pacing. Results are shown for different pacing locations relative to scar, for the 6 different FEC setups described in Table S1.

1. **Comparison between endocardial and epicardial pacing in absence of a transmural APD gradient**

Figure 4 in the manuscript shows a significant difference between the volumes of HRG during endocardial and epicardial pacing in the absence of a transmural APD gradient. However, this difference was not seen in the idealised models (Figure 6). When pacing the heart, high repolarization gradients are created near the pacing site and expand in the direction orthogonal to the fibre orientation, as shown in our previous study (Figure 3; Mendonca Costa et al 2019^2^). Because of transmural fibre rotation and reduced electrotonic load close to the opposite surface, HRG are seen intramurally but these disperse as the wave reaches the opposite surface. Thus, there is a larger HRG volume close to the pacing surface than at the opposite surface. Moreover, the epicardium has a larger surface area than the endocardium and most scars in our models are not fully transmural, which means more viable tissue on the epicardium than on the endocardium surface. Thus, the repolarization is allowed to propagate through a larger volume of tissue just below the epicardium than on the endocardium, where propagation is blocked by the scar. These two effects are illustrated in Figure S2. Here, when pacing at the epicardial surface, the HRG expand further on the epicardial surface during epicardial pacing (Figure S2-D) than on the endocardial surface during endocardium pacing (Figure S2-A). This effect is particularly evident when focusing on the regions highlighted by the pink circles. In addition, the volume of HRG is higher at the pacing surface than on the opposite surface.

To quantify this effect, we computed the volume of viable tissue and of HRG within 1cm from scar on the endocardium and epicardium (within 1mm below the surface) during endocardial and epicardial pacing. As shown in Figure S3, a larger volume of viable tissue is present on the epicardium than on the endocardium, owing to a larger surface area and less scar. In addition, as shown in Figure S4, the volume of HRG is significantly larger in the epicardium than on the endocardium during both endocardial (light red) and epicardial (black) pacing and for all pacing locations. As before, the difference in volume of HRG between pacing locations during epicardial pacing is significant between pacing locations 0.2 and 2.5cm and between 0.2 and 3.5cm, but these not significantly change during endocardial pacing. These effects are not captured in our idealised models, as these have a transmural scar and both surfaces have the same surface area. This, thus, explains the discrepancy in volume of HRG during endocardial and epicardial pacing between the idealised and patient models.


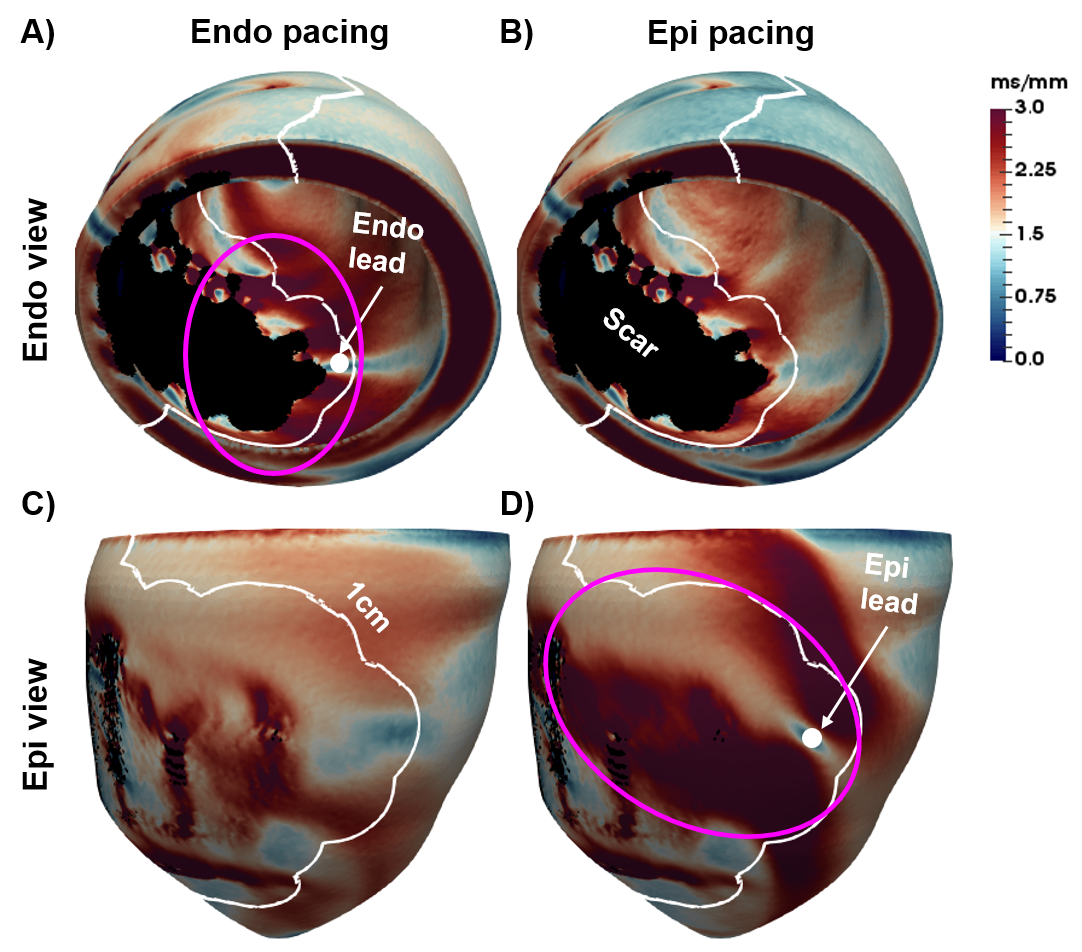


Figure S2: Spatial distribution of repolarization gradients during endocardial (left) and epicardial (right) pacing. The scar is shown in black. The white isolines indicate the region within 1cm from the scar. Endocardial and Epicardial lead locations are indicated by the white filled circles. The pink circles indicate areas within 1cm from the scar with high repolarization gradients (dark red).


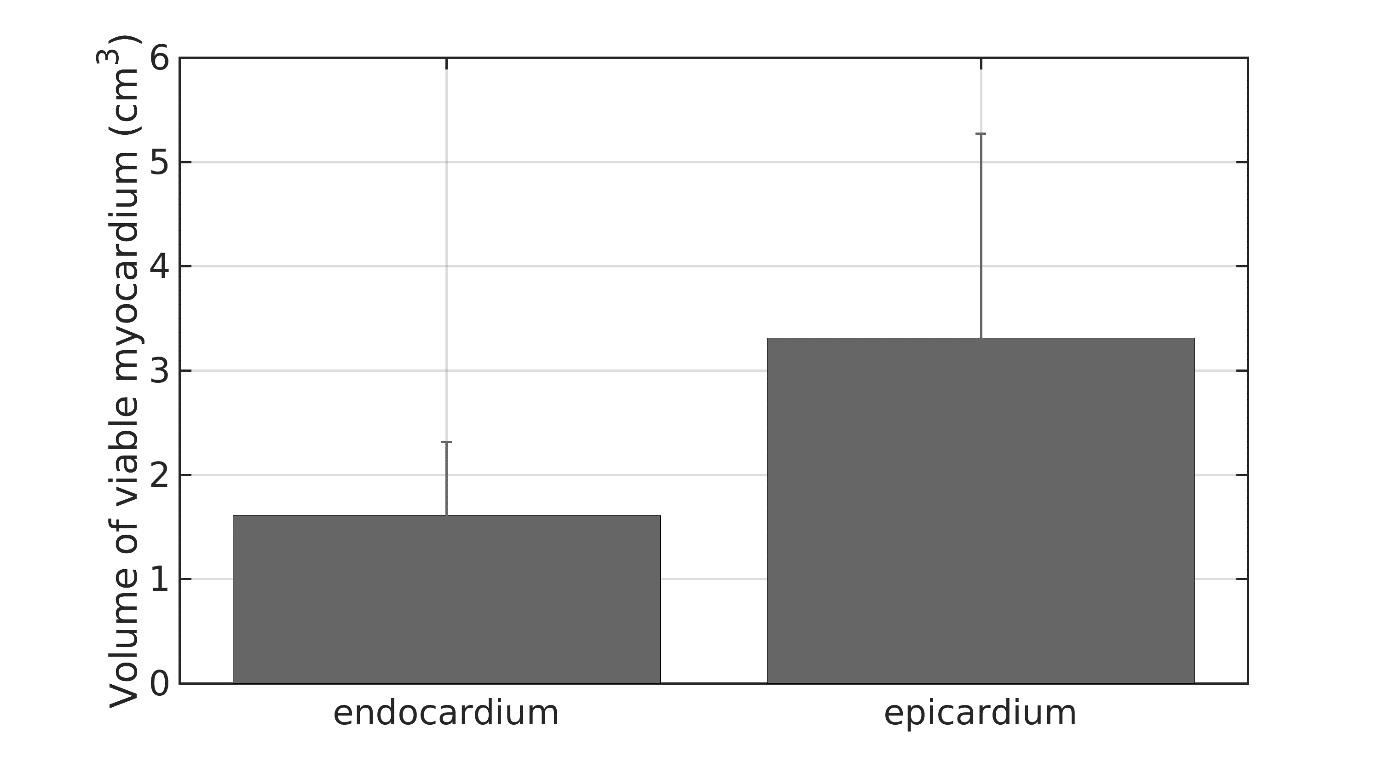


Figure S3: Mean volume of viable tissue on the endocardium and epicardiaum within 1mm below the surface across all patient models.


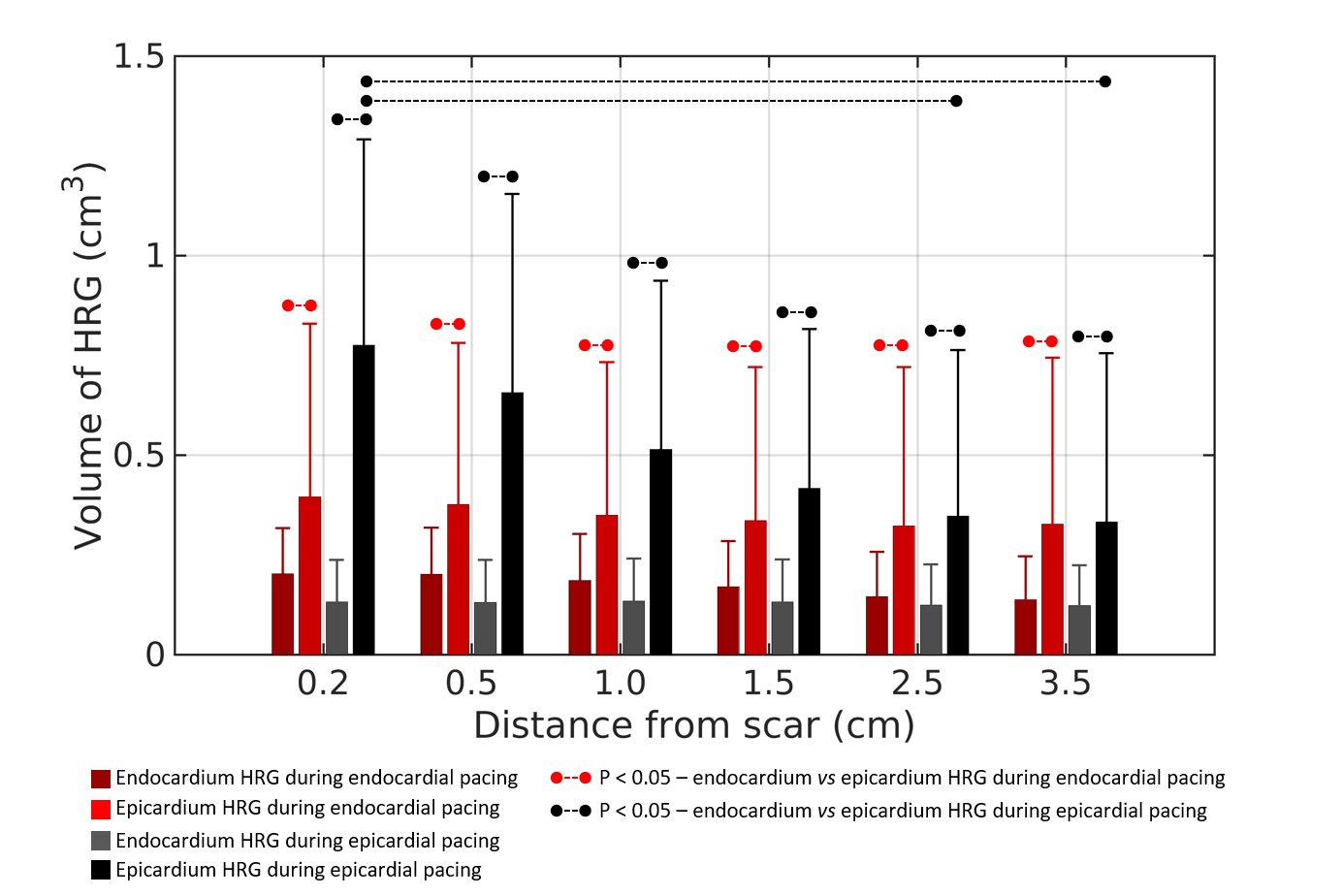


Figure S4: Volumes of high repolarization gradients (HRG) at the endocardium (dark red) and epicardium (light red) during endocardium pacing and at the endocardium (dark grey) and epicardium (black) during epicardium pacing. Comparisons between the endocardium (dark and light red) and epicardium (grey and black) HRG and between pacing locations are shown by the red and black lines for endocardial and epicardial pacing, respectively.

**References**

1. Sano T, Takayama N, Shimamoto T. Directional difference of conduction velocity in the cardiac ventricular syncytium studied by microelectrodes. *Circ Res*. 1959;7(2):262-267. doi:10.1161/01.RES.7.2.262

2. Mendonca Costa C, Neic A, Kerfoot E, et al. Pacing in proximity to scar during cardiac resynchronization therapy increases local dispersion of repolarization and susceptibility to ventricular arrhythmogenesis. *Hear Rhythm*. 2019;16(10):1475-1483. doi:10.1016/j.hrthm.2019.03.027
